# Visual word recognition of Chinese–Japanese bilinguals: limited role of phonology

**DOI:** 10.3389/fpsyg.2023.1318798

**Published:** 2024-01-04

**Authors:** Zhongyan Jiao, Leyi Du, Yifan Wang, Yixian Li

**Affiliations:** ^1^School of Foreign Language, Ningbo University of Technology, Ningbo, China; ^2^School of Sciences, Ningbo University of Technology, Ningbo, China

**Keywords:** masked priming effect, phonological priming, ERP, Chinese–Japanese bilinguals, logographic scripts

## Abstract

**Introduction:**

The investigation of how orthography and phonology influence lexical semantic access in visual word identification is a crucial area in psycholinguistics. Previous studies, focusing on alphabetic scripts in bilingual lexical recognition, have highlighted the facilitative role of phonological similarity. Yet, the impact of cross-language phonological similarity in bilinguals using non-alphabetic scripts remains unclear.

**Methods:**

In this study, we employed a lexical decision task to examine Chinese–Japanese bilinguals. Participants were presented with Chinese–Japanese cognate translation pairs, categorized into phonologically similar and dissimilar cognates.

**Results:**

Analysis of event-related potentials (ERP) revealed no significant differences between phonologically similar and dissimilar contexts in the early time windows (90–170 ms and 170–270 ms). However, in the later time window (350–500 ms), significant differences were observed, with the phonologically dissimilar condition eliciting a larger negative wave.

**Discussion:**

Contrary to findings in alphabetic script-based studies, our results suggest that in logographic script processing, the activation of phonology and semantics occurs simultaneously, and the influence of phonology is limited. This indicates a distinct cognitive processing mechanism in non-alphabetic language bilinguals, providing new insights into the dynamics of bilingual lexical recognition.

## 1 Introduction

In the field of bilingualism, there has been much interest in the way bilinguals access and process lexical-semantic expressions. According to the orthographic, phonological, and semantic notations of the bilingual interactive activation (BIA) model and BIA plus (BIA+) model, the orthographic, phonological, and semantic expressions of two languages were said to be intermingled and connected in bilinguals’ lexicons (Dijkstra and van Heuven, 2002). In case bilinguals read in their second language, there would be a bottom-up collaborative co-activation of lexical notations from the first language, according to a wealth of evidence from researches with interlingual homographs/ homophones or cognate words (Dijkstra et al., 1999; Lemhofer and Dijkstra, 2004; Voga and Grainger, 2007; Davis et al., 2010; Duñabeitia et al., 2010; Nakayama et al., 2013, 2014). This argument is based on the observation that identification of target words in a second language is typically facilitated by a masked prime that is briefly expressed in the first language.

According to researches concerning alphabetic scripts, phonology exerts a significant facilitating action on bilingual lexical recognition, regardless of whether the two languages share the same script (Voga and Grainger, 2007; Carreiras et al., 2009; Jouravlev et al., 2014). Recently, an increasing number of researchers have begun to emphasize phonological priming research in logographic lexical recognition.

Several studies have examined cross-script phonological facilitation between two languages, one of which is logographic. In Zhou et al. (2010), in both lexical decision and naming tasks, faster response of Chinese-English bilinguals to English targets (e.g., “door”) was noted upon precedent exposure to phonologically associated one-character Chinese primes (e.g., 道/dao4/) when compared to precedent exposure to irrelevant Chinese words. Ando et al. (2014) performed a masked phonological priming task, where English targets and logographic Japanese kanji primes were utilized to determine if Japanese-English bilinguals have integrated phonological memories for respective languages. In their trials, phonologically identical kanji primes aided in making lexical judgments for English target words. Chinese-English cognate words were used in Zhang et al. (2019) masked priming experiment with a lexical judgment task in L1 (Chinese)-L2 (English) along with L2-L1 orientations, demonstrating creation of a large priming effect by L2-L1 cognates (English words) compared to noncognates (English words), whereas absence of such an effect by L1-L2 cognates (Chinese words) or noncognates (Chinese words). They concluded that the writing system modifies the cognate facilitation priming effect.

However, there appeared phonological facilitation for bilinguals with respective languages containing a minimum of single alphabetic language in all the above researches. Phonological activation is believed to be largely automatic when words are recognized in alphabetic languages. Thus, when at least one of the bilinguals’ languages is alphabetic, it follows that target identification would be facilitated as a result of phonological resemblance between primes and targets attributed to the importance of phonology played in those languages. However, the question that remains unanswered is whether cross-language phonological similarities facilitate target processing for bilinguals with logographic languages. To date, only one relevant study has been identified. Liu et al. (2023) (in their Experiment 1) examined Chinese–Japanese bilinguals using same-script cognates (Chinese and Japanese kanji) as primes and targets with a masked word naming task. They tested the reaction time, and behavioral data showed no significantly varying extents of priming effects across phonologically analogous (e.g., 信赖/xin4lai4/−信頼/shinrai/) and disparate cognate pairs (e.g., 保证/bao3zheng4/−保証/houshou/), indicating the absence of phonological similarity effect. From the studies of Zhang et al. (2019) and Liu et al. (2023), it appears that phonological similarity has little impact on the processing of logographic scripts (e.g., Chinese), contrasting with findings from alphabetic script research.

The present study intends to focus on Chinese-Japanese bilinguals employing ERP technology. Through maneuver of phonological similarity among Chinese–Japanese cognate words, we aim to examine whether visual lexical processing of bilinguals who only use logographic scripts (such as Chinese and Japanese kanji) is affected by such similarity, further exploring the nature of bilingual lexical expression.

Chinese-Japanese cognates were used as critical stimuli. As Japanese kanji characters were borrowed from Chinese, the readings of many kanji characters reflect the readings of the Chinese characters when they were integrated into Japanese. Meanwhile, the readings of some Chinese characters have since changed in China, whereas the original readings have typically been retained in Japan (Liu et al., 2023). Therefore, in Chinese-Japanese cognates, some words are phonologically very similar (e.g., 安心/an1xin1/−安心/ansin/), while others are phonologically quite dissimilar (e.g., 条件/tiao2jian4/−条件/jyouken/), even though they share the same orthography and semantics.

We fulfilled the lexical decision task by employing L1-L2 direction masked priming paradigm and recorded participants’ brainwaves. In relevant experiments, a masked priming paradigm is usually adopted in lexical decision tasks (Holcomb and Grainger, 2006; Okano et al., 2013; Jouravlev et al., 2014; Zhang et al., 2019). According to Forster and Davis (1984), in this paradigm, a prime is presented for a very short duration (typically 50–60 ms) and is masked, making most participants unaware of the prime’s presence. Thus it can ensures that the experimental results more genuinely reflect word processing. In our experiment, to ensure unconscious processing of the prime by participants, the following design was employed: (1) The prime word was presented for 47 ms; (2) All stimuli were centrally presented, and considering the stimuli were two-character Chinese words, we used four hash marks (####) to make the mask completely obscured the prime stimulus; (3) The prime stimulus and the target stimulus were used for different fonts and points. We employed a forced-choice test to evaluate the success of the masking. In which participants were shown masked words after the main experiment and were required to make explicit forced-choice decisions regarding each stimulus to determine if they perceived the masked words. An objective identification threshold was set at a 50% accuracy rate (Dehaene et al., 2001; Etkin et al., 2004).

Since research based on behavioral outcome measures (e.g., reaction time and accuracy) is hardly to separate the specific processing of orthography, phonology, and semantics in word recognition, while Event-related potential (ERP) technology, with precise temporal resolution, can distinguish between morphological and semantic processing in language. Holcomb and Grainger (2006) used ERP technology to study the word identification process and discovered a set of ERP constituents (N250, N400, and N/P150) that are very susceptible to masked repetition priming. The amplitude of these components is modulated by priming stimuli, reflecting the entire processing from orthographic representation (N/P150) to phonological representation (N250) and finally semantic access (N400).

The hypothesis of our experiment is that if phonological similarity facilitates the lexical processing of the second language in bilinguals using logographic language, then in behavioral data, the reaction times (RTs) for phonologically similar cognates will be significantly shorter than those for phonologically dissimilar ones; in the ERP data, the N250 effect will be observed. The N250 has a wide distribution, and occurs in the case of less target–prime form superpositionis, report Holcomb and Grainger (2006). This component links to orthographic and phonological processing in the mental lexicon and has been supported by additional studies (e.g., Holcomb and Grainger, 2007; Okano et al., 2013). Given that prior research (Liu et al., 2023) suggests a weaker phonological activation for logographic scripts, Chinese-Japanese bilinguals may not exhibit cross-linguistic phonological facilitation. Whether such an effect can be observed will depend on the experimental outcomes.

## 2 Materials and methods

### 2.1 Participants

Twenty eight juniors majoring in Japanese, of whom 19 were females and 9 were males, participated in this experiment. They were 21.5 (SD = 1.5) years of age on average. All participants’ native language was Chinese, and they could speak standard Mandarin. They had been studying Japanese for 3 years and had no exposure to Japanese before entering university. All participants passed the Japanese Language Proficiency Test (JLPT) N2 exam. The entire subjects were right-handed, whose vision was either normal or rectified to normal. This was the first time they had participated in such an experiment, having signed informed consent forms before the experiment began. In return, they were remunerated for their part.

### 2.2 Stimuli

Initially, 300 pairs of cognate translation equivalents (with identical orthography and essentially the same semantics) were selected from “Chinese Corresponding to Japanese” (Japan Cultural Agency, 1978). Twenty five additional bilinguals who were proficient in both Chinese and Japanese and had not been involved in the experiment, were asked to assign a score from 1 (not similar at all) to 5 (perfectly similar) to the 300 pairs of cognate translation equivalents according to their level of phonological similarity, as well as Familiarity on a scale from 1 (not familiar at all) to 7 (very familiar). Based on these scores, 60 pairs of phonologically similar words (phonological similarity: 3.32 ± 0.12, familiarity: 6.52 ± 0.12, number of strokes: 14.58 ± 4.22) and 60 pairs of phonologically dissimilar words (phonological similarity: 2.07 ± 0.25, familiarity: 6.43 ± 0.33, number of strokes: 14.20 ± 3.67) were chosen. A significant disparity in phonological similarity was noted between the two cognate types (*t*(118) = 35.38, *p* < 0.001), while no significant difference was noted in familiarity (*t*(118) = 1.83, *p* = 0.07).

Pairing of a total of 120 targets was accomplished with 2 categories of Chinese primes: cognates and unrelated words. Phonologically similar cognates match the target words in orthography, phonology, and semantics (e.g., 安心/an1xin1/−安心/ansin/). Phonologically dissimilar cognates match the target words in orthography and semantics (e.g.,条件/tiao2jian4/−条件/jyouken/). Unrelated words differ from the target words in orthography, pronunciation, and semantics (e.g.,少女/shao4nv3/−安心/ansin). The source of these unrelated words was “Modern Chinese Dictionary (7th Edition)” (Institute of Linguistics and Chinese Academy of Social Sciences, 2016) with a mean stroke number of 14.26 ± 3.73. To maintain balance, another 120 Japanese nonwords (mean stroke number 13.98 ± 3.59) were adopted. Considering the orthographic resemblances between Chinese and Japanese cognates, to ensure that participants engaged in a Japanese linguistic context, the selected target nonwords were valid in Chinese but invalid in Japanese, serving as a check for manipulation. These Japanese nonwords were then matched with 120 Chinese cognate primes (mean stroke number 13.98 ± 3.59) and 120 Chinese unrelated primes (mean stroke number 13.81 ± 3.61). The mean stroke number for the three types of target words (phonologically similar, phonologically dissimilar, and nonwords) showed no significant difference (*F*(2,357) = 0.64, *p* = 0.53). The mean stroke number for the four types of prime words (phonologically similar, phonologically dissimilar, unrelated, and nonwords) also showed no significant difference (*F*(3,476) = 0.89, *p* = 0.45).

The experimental materials were divided into 6 blocks using a Latin square design. Each block contained all experimental conditions and was pseudorandomly balanced. Each block was presented randomly.

### 2.3 Procedure

Materials were programmed using E-prime (3.0) and presented using white font against a black background via a desktop application. Following the convention in psychology, the presentation of targets and primes was accomplished using different categories and lengths of fonts: the targets were presented in 14 pt. in Japanese font (MS Mincho), whereas the primes were presented in 12 pt. in Chinese font (SimSun).

**Figure 1:**
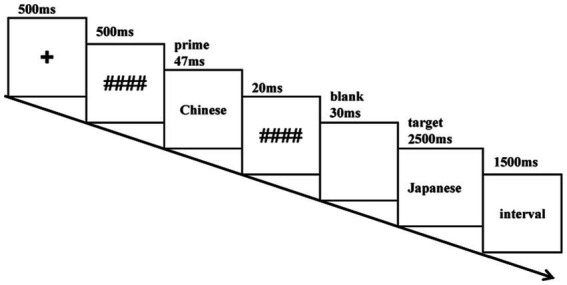
Procedure for one trial.

The trial sequence description is given below (Figure 1). After the display of a fixation point “+” at the screen center for 500 ms, a forward mask (####) is presented for 500 ms and then the prime Chinese word is shown for 47 ms. A 20 ms presentation of the backward mask (####) is followed by a 30 ms display of a blank screen, and subsequently target Japanese word is presented for 2,500 ms, waiting for the subjects’ key response. In case a subject failed to respond within 2,500 ms, the screen vanished automatically, and the response was documented as incorrect. The experiment involves a lexical decision task, during which subjects are required to make a quick and accurate judgment upon seeing the target Japanese word. Participants press the “F”-key in the case of nonwords, whereas the “J”-key in the case of real words. Before the experiment, there was a practice phase to ensure that participants were fully familiar with the experimental procedure. In the practice section, there were 24 pairs of trials, comprising 12 pairs of real words and 12 pairs of nonwords. Participants were required to achieve an accuracy rate of 85% to proceed to the main experiment, since a lower accuracy rate might indicate that the participant was still in monolingual Chinese mode. Finally, an additional forced-choice test was implemented, focusing on primes with the same parameters as those used in the main experiment. In this variation, targets were omitted, and participants were required to select between two words presented to the left and right of the fixation point. The entire experiment took approximately 40 min.

### 2.4 Data collection and analysis

An actiCHamp amplifier (Brain Products GmbH, Germany) was utilized to acquire EEG data from 32 Ag/AgCl active electrodes (10–20 system). Online filtration of data was accomplished through a bandpass filter (0.05–100 Hz), while the data sampling frequency was 500 Hz. The left mastoid served as the reference electrode. Impedances of scalp electrode were maintained below 5 kΩ.

Data assessment was accomplished by running EEGLAB (Delorme and Makeig, 2004) and ERPLAB (Lopez-Calderon and Luck, 2014) toolboxes on MATLAB 2018b (Mathworks, Natick, MA, United States). After eliminating bad segments and interpolating bad traces, the right mastoid was chosen as the re-referencing electrode, and the bandpass was filtered between 0.1 and 30 Hz.

The Independent component analysis (ICA) approach was adopted to accomplish the mathematical rectification of ocular artifacts. Following target onset, segmentation of data proceeded between −200 and 800 ms. Before the onset of target stimuli, baseline rectification was undertaken between −200 ms and 0 ms. We included segments whose maximally permissible amplitude was ±80 μV. Averaging of ERP waveforms was accomplished according to experimental conditions. Finally, the ERP waveforms for all correct trials across different conditions were averaged to obtain the grand average for each participant.

Based on previous research (Holcomb and Grainger, 2006; Grainger and Holcomb, 2009; Okano et al., 2013) and observations of the grand average wave, the average amplitudes of the N150, N250, and N400 components were analyzed. The time window for the N150 component was 90–170 ms after stimulus presentation, the N250 component was 170–270 ms, and the N400 component was 350–500 ms.

The SPSS 20.0 software was deployed for the relevant data analyses. For reaction time and accuracy, two separate two-way repeated-measures ANOVAs were carried out based on phonological similarity (similar vs. dissimilar) × prime type (cognate vs. unrelated). In terms of ERP data, a three-way repeated-measures ANOVA was applied for each time window, contrasting phonological similarity (similar vs. dissimilar) × prime type (cognate vs. unrelated) × electrode as within-subject factors. Analyses were first conducted with electrode sites (F3, F4, Fz, C3, C4, Cz, P3, P4, Pz, O1, O2, Oz), and then were performed separately for three groups of electrodes, in the left (P3,C3,P7,CP5), center (PO7,PO8,O1,O2,Oz), and right (P4,C4,P8,CP6). The Greenhouse–Geisser correction was applied where appropriate, and Bonferroni corrections were applied for pairwise comparisons.

## 3 Results

### 3.1 Behavioral data

Three participants had an accuracy rate lower than 80%, and the data from these 3 participants were excluded. Correct responses with RTs between 300 and 1700 ms were analyzed, causing the exclusion of 14.8% of the total data. In the forced-choice test, All of 25 included participants performed at or below chance level. The observed success rate of 49.3% was not statistically different from the 50% success rate expected by chance (*t*_24_ = 1.80, *p* = 0.08).

The ANOVA on RTs showed that the main effect of phonological similarity was not significant, *F*(1, 24) = 2.065, *p* = 0.164, *ηp*^2^ = 0.079. The main effect of prime type was of significance, *F*(1, 24) = 209.944, *p* < 0.001, *ηp^2^* = 0.897, showing longer RTs for unrelated primes compared to cognate primes (*M* = 94.820, *SEM* = 6.544, *p* < 0.001). The interaction between phonological similarity and prime type was not of significance, *F*(1, 24) = 2.565, *p* = 0.122, *ηp*^2^ = 0.097.

**Figure 2:**
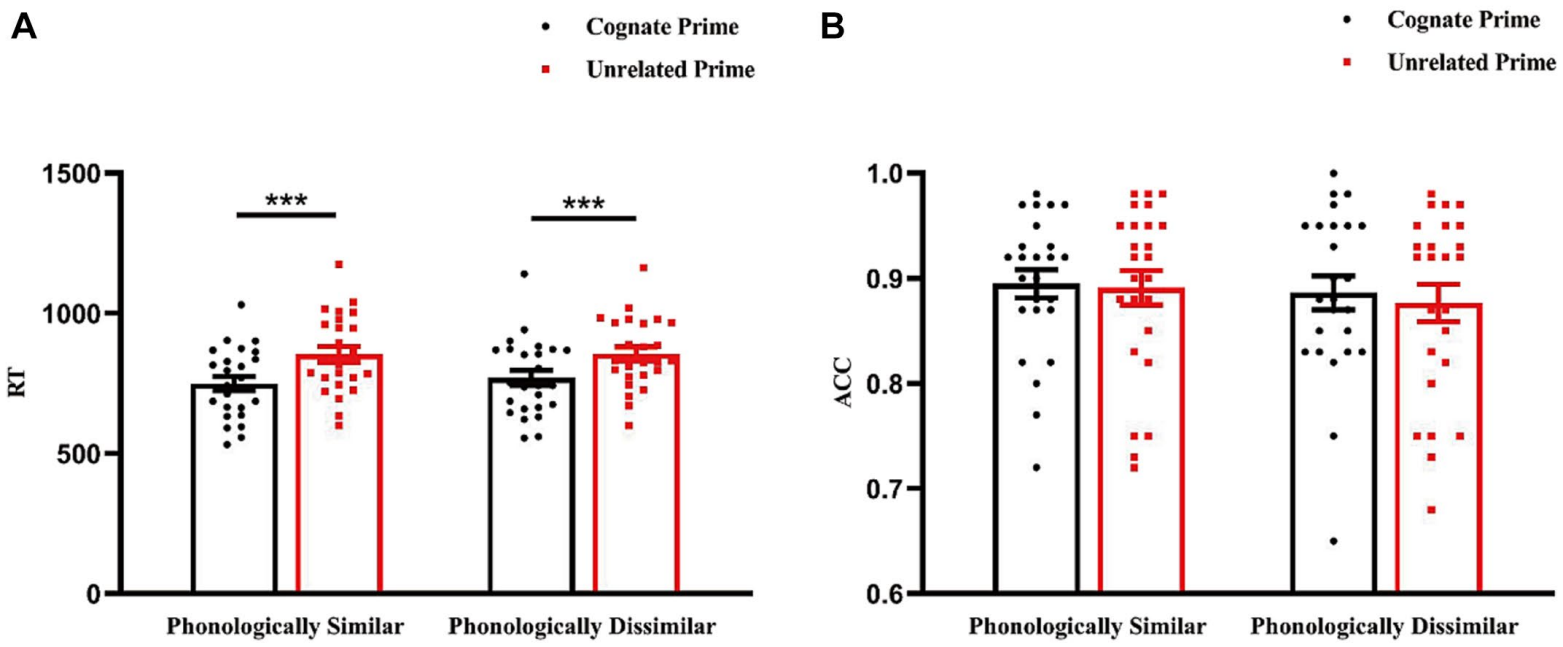
Reaction time **(A)** and accuracy rate **(B)** across the conditions. Error bars represent standard error of mean. ***p < 0.001.

For accuracy rates(ACC), the analysis demonstrated that the main effect of phonological similarity was not significant, *F*(1, 24) = 0.738, *p* = 0.399, *ηp*^2^ = 0.030. The main effect of prime type was not of significance, *F*(1, 24) = 1.049, *p* = 0.316, *ηp*^2^ = 0.042. The interaction between phonological similarity and prime type was not of significance, *F*(1, 24) = 0.282, *p* = 0.601, *ηp*^2^ = 0.012 (Figure 2).

### 3.2 ERP data

**Figure 3:**
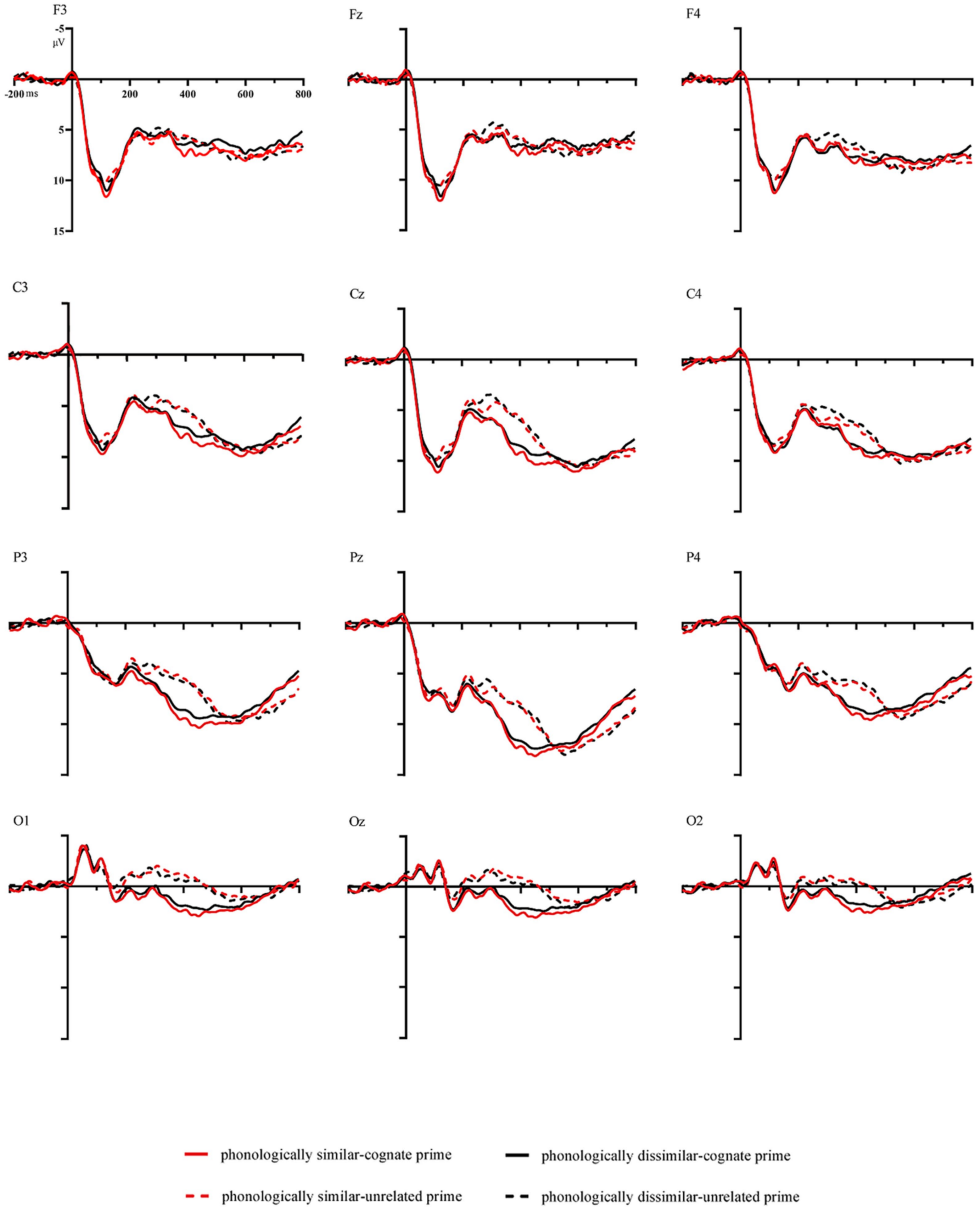
Grand average waveforms in the different conditions by electrode.

Data from the three participants whose accuracy rate lower than 80% was excluded. Figure 3 presents the waveforms evoked in response to targets in different conditions by electrode.

Epoch analyses of the ERP data

#### 3.2.1 90–170 ms window

During this time window, prime type presented a significant main effect, *F*(1, 24) = 5.145, *p* = 0.033, *ηp*^2^ = 0.177. Phonological similarity had no significant main effect. *F*(1, 24) = 2.124, *p* = 0.158, *ηp*^2^ = 0.081. There existed a significant main effect of electrode sites, *F*(2, 47) = 88.521, *p* < 0.001, *ηp*^2^ = 0.787. In addition, a significant interaction between prime type and electrode sites was also observed, *F*(4, 93) = 8.109, *p* < 0.001, *ηp*^2^ = 0.253, showing more positive amplitudes for cognate primes than for unrelated primes at the Fz, F3, C3, Cz, C4, and F4 sites. Other interactions were not significant (*ps* ≥ 0.158). In the analyses by regions, only a significant main effect of regions was found, *F*(1, 29) = 26.863, *p* < 0.001, *ηp*^2^ = 0.528. No other significant effects were shown in this time window (*ps* ≥ 0.210).

#### 3.2.2 170–270 ms window

In the analysis with electrodes, ANOVA showed a significant main effect of prime type, *F*(1, 24) = 8.178, *p* = 0.009, *ηp*^2^ = 0.254. The main effect of phonological similarity was not of significance, *F*(1, 24) = 0.572, *p* = 0.457, *ηp*^2^ = 0.023. There was a significant main effect of electrode sites, *F*(2, 51) = 30.767, *p* < 0.001, *ηp*^2^ = 0.562. In addition, a significant interaction between prime type and electrode sites was also found, *F*(2, 54) = 5.702, *p* = 0.004, *ηp*^2^ = 0.192, showing more negative amplitudes for unrelated primes than for cognate primes at the Pz, P3, P4, O1, Oz, O2, C3, Cz, and C4 sites. Other interactions were not significant (*ps* > 0.108).

In the analysis by regions, there was a significant main effect of region [*F*(2, 48) = 29.263, *p* < 0.001, *ηp*^2^ = 0.549] as well as prime type[*F*(1, 24) = 17.877, *p* < 0.001, *ηp*^2^ = 0.453]. The main effect of phonological similarity was not of significance, *F*(1, 24) = 0.523, *p* = 0.477, *ηp*^2^ = 0.021. A significant interaction between prime type and region was also found, *F*(2, 48) = 14.460, *p* < 0.001,*ηp*^2^ = 0.376, showing more negative amplitudes for unrelated primes than for cognate primes at the central region and the right region. Other interactions were not significant (*ps* ≥ 0.151).

#### 3.2.3 350–500 ms window

Analysis with electrodes showed a significant main effect of prime type [*F*(1, 24) = 48.481, *p* < 0.001, *ηp*^2^ = 0.669] and electrode sites [*F*(2, 43) = 35.976, *p* < 0.001, *ηp*^2^ = 0.600]. A significant interaction between prime type and electrode sites was also found, *F*(2, 49) = 12.910, *p* < 0.001, *ηp*^2^ = 0.350, showing more negative amplitudes for unrelated primes than for cognate primes at the Pz, P3, P4, O1, Oz, O2, C3,Cz, and C4 sites. Other effects were not significant (*ps* ≥ 0.056).

**Figure 4:**
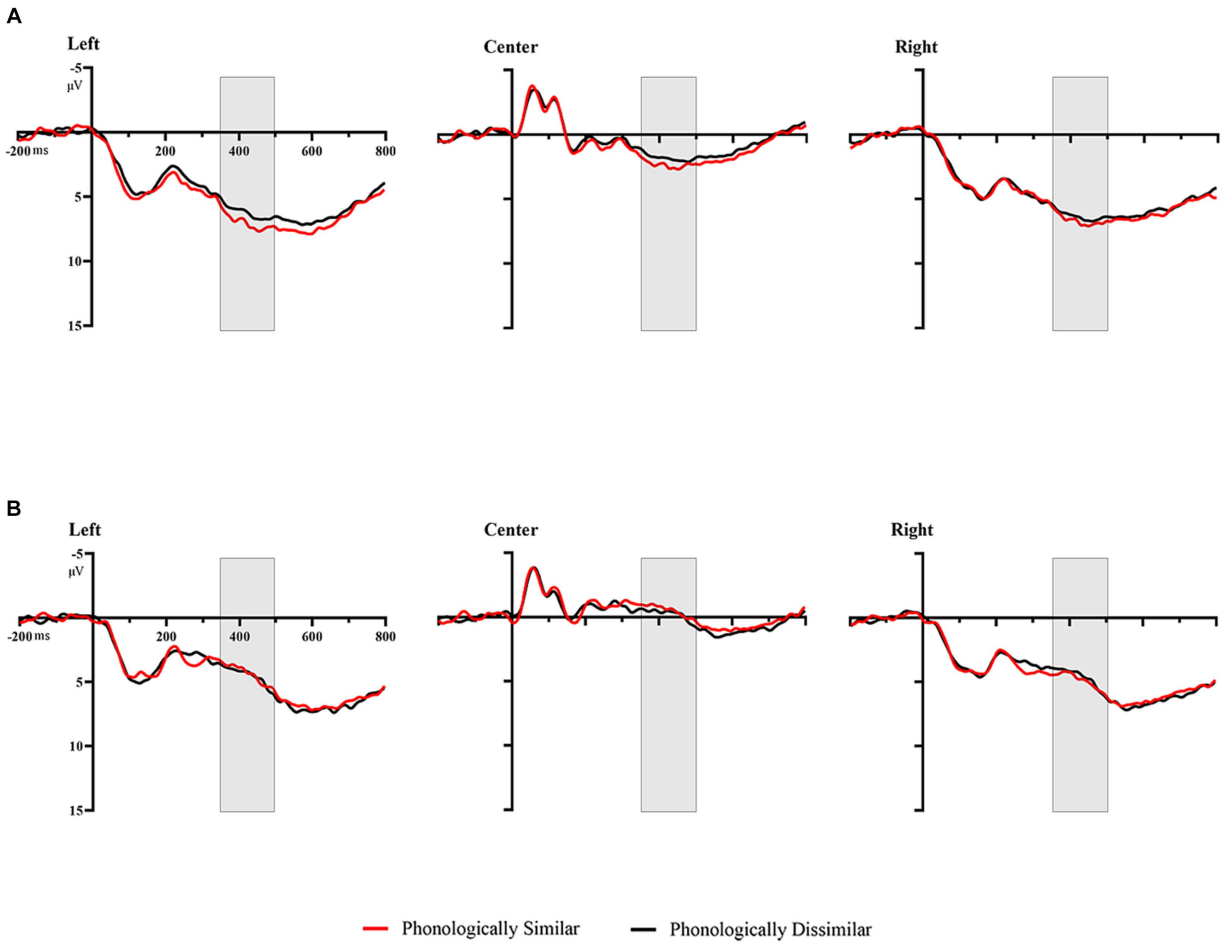
Grand average waveforms in the different conditions by region. **(A)** Phonologically similar vs. phonologically dissimilar when the prime was cognate. **(B)** Phonologically similar vs. phonologically dissimilar when the prime was unrelated.

**Figure 5:**
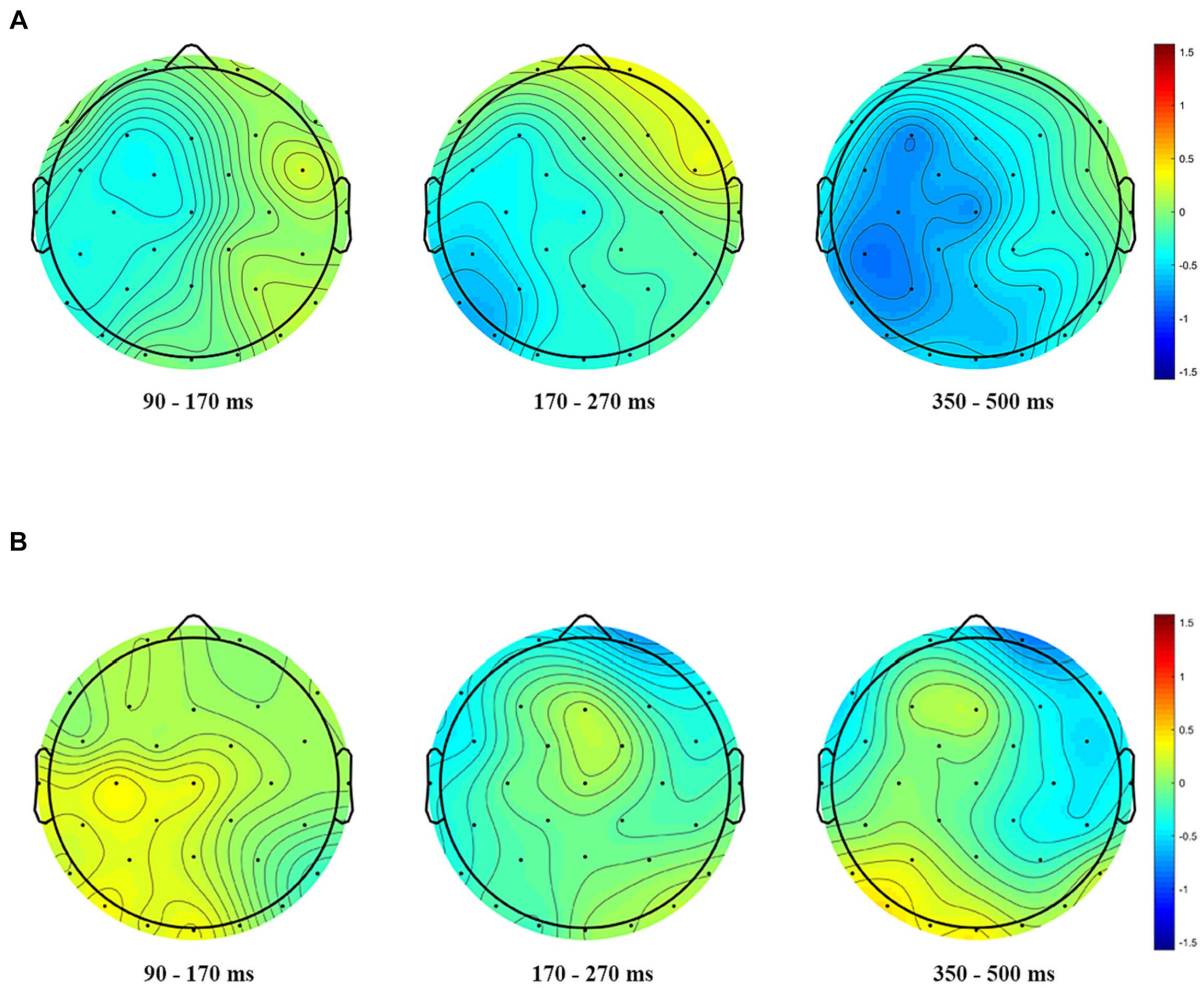
Topographical distribution of the phonological priming effects (phonologically dissimilar – phonologically similar) in the three windows of analyses. **(A)** Phonological priming effects when the prime was cognate. **(B)** Phonological priming effects when the prime was unrelated.

In the analysis by regions, there existed a significant main effect of prime type [*F*(1, 24) = 171.488, *p* < 0.001, *ηp*^2^ = 0.887]as well as phonological similarity [*F*(1, 24) = 4.822, *p* = 0.038,*ηp*^2^ = 0.167] and region [*F*(2, 48) = 89.424, *p* < 0.001, *ηp*^2^ = 0.788]. A significant three-way interaction among phonological similarity, prime type and the region was also found, *F*(2, 48) = 4.309, *p* = 0.019, *ηp*^2^ = 0.152. Further analyses showed that when the prime was cognate, phonologically dissimilar primes elicited more negative amplitudes compared to phonologically similar primes at left and center regions; and when the prime was unrelated, phonologically similar primes and phonologically dissimilar primes showed no differences(*ps* ≥ 0.406) (Figures 4, 5).

To summarize, the RTs data suggested an orthographic effect but no phonological effect. However, the ERP data revealed orthographic effect in the 90–170 ms and170-270 ms time windows, wherever phonological effect in the 350–500 ms time window.

## 4 Discussion

Exploring the effects of L1-L2 phonological priming in Chinese–Japanese bilinguals, a masked priming paradigm and EEG recordings were employed in this study. Participants were tasked with making a lexical judgment on phonologically similar cognates and phonologically dissimilar cognates with different types of prime. The behavioral results demonstrated that there existed a significant difference between cognate priming and unrelated priming, while the difference between phonologically similar primes and phonologically dissimilar primes was not of significance. Regarding ERP results, a significant difference was found between cognate priming and unrelated priming within the 90–170 ms and 170–270 ms window, whereas the difference between phonologically similar cognates and phonologically dissimilar cognates was not significant in the 170–270 ms window, suggesting a limited role of phonology in logographic scripts processing. These findings stand in contrast to studies on alphabetic scripts and syllabary scripts (e.g., Grainger et al., 2006; Nakayama et al., 2012; Okano et al., 2013; Jouravlev et al., 2014; Ando et al., 2015) but are in line with a behavioral study where no phonological priming effect was found in Chinese-Japanese bilinguals (Liu et al., 2023).

### 4.1 Time course of logographic script processing

In our results, we identified the ERP components frequently mentioned in visual lexical processing: N/P150, N250, and N400 (Holcomb and Grainger, 2006; Grainger and Holcomb, 2009; Jouravlev et al., 2014; Xiong et al., 2020; Chen et al., 2021). The N/P150 component (depending on the electrode location) reflects the cognitive process of mapping visual features onto prelexical orthographic representations. In contrast, the N250 effect has been caused by a variety of prelexical processes, including mapping prelexical form representations onto whole-word form representations, as well as orthographic to phonological mapping. As a late ERP component, N400 has been regarded to be an index of semantic processing (Shetty et al., 2022).

The current work showed that within the 90–170 ms time window, the amplitude induced by cognate primes was significantly more positive than that induced by unrelated primes, indicating that in the 90–170 ms time window, orthographic information was activated first, aligning with previous research conclusions from alphabetic scripts. Within the 170–270 ms time window, the amplitude elicited by unrelated primes was more negative than that elicited by cognate primes, whereas no significant difference between the phonologically similar primes and phonologically dissimilar primes was found, suggesting that the N250 component here still reflects orthographic processing, which differed from the conclusions of alphabetic script studies. Studies on alphabetic scripts have shown a significant difference between phonologically similar and phonologically dissimilar conditions, leading to the belief that N250 reflects phonological processing (Jouravlev et al., 2014; Ando et al., 2015). Within the 350–500 ms time window, the amplitude elicited by unrelated primes was more negative than that by cognate primes, suggesting the activation of semantics. Moreover, we observed a more negative amplitude elicited by phonologically dissimilar cognate primes than by phonologically similar cognate primes from the parietal region, suggesting that phonological information was fully activated, and led to a phonological effect. The result implies that phonology and semantics were activated almost at the same time in logographic scripts, which also verified why in the behavioral data, the RTs for both types of cognate primes showed almost no difference.

### 4.2 Phonological priming in logographic script processing

In addition, the obtained results also offer information in terms of whether the priming effect can be produced based only on interlingual phonological similarity in Chinese-Japanese bilinguals. To our knowledge, the present research is the first to discover the possibility of phonological priming in logographic scripts, as shown in both participants’ RTs and their ERP responses.

Several studies have explored cross-script phonological priming with the use of alphabetic or syllabary scripts. In one such study, an experiment by Voga and Grainger (2007) revealed that Greek-French bilinguals responded more quickly to French words when preceded by primes which contained both semantic and phonological similarities, as opposed to primes which had only semantic similarity. Jouravlev et al. (2014) found that for Russian-English bilinguals performing naming tasks in the L2 (English) to L1 (Russian) direction, RTs under phonological overlap conditions were shorter than in unrelated conditions. Moreover, ERP data also indicated smaller amplitudes elicited under phonological overlap conditions compared to unrelated conditions. In another relevant study, Nakayama et al. (2014) demonstrated that the magnitude of the priming effect is influenced by the degree of phonological overlap between cognate translation equivalents. Ando et al. (2015) revealed that Japanese-English bilinguals displayed faster naming latencies for English target words preceded by phonologically related Katakana primes, in contrast to unrelated Katakana primes. These research findings suggest that phonology facilitates the lexical access of bilinguals in alphabetic languages. However, our study found that for phonologically similar and dissimilar cognate primes, the RTs do not differ significantly (749 ms vs.769 ms). The ERP findings also highlight that the activation times for phonology and semantics are similar, indicating that phonology has a constrained role in bilingual lexical processing.

This study’s discoveries largely coincide with the conclusions of two other investigations on phonological priming in logographic scripts (Wong et al., 2014; Liu et al., 2023). Wong et al. (2014) used a lexical decision task, exploring the influence of four prime types: orthographically related, phonologically related, semantically related, and unrelated. They examined the impact of phonology on the comprehension of Chinese compound words, finding that both orthographically and semantically related conditions elicited notable ERP effects, while the phonologically related condition did not show significant variations. Liu et al. (2023) employed a word naming task, contrasting the priming effects between phonologically similar and dissimilar Chinese-Japanese cognate pairs. They observed no significant difference in the priming effects (69 ms vs. 64 ms). Based on this, they concluded that there is no effect of interlingual phonological similarity.

As noted, in Wong et al.’s (2014) experiment, no significant differences were observed under phonologically similar conditions. However, in the current study, significant differences emerged under phonologically similar conditions within the 350–500 ms time window. The discrepancy in outcomes could be the result of the materials employed in the experiments. Wong et al. (2014) employed initial-related pairs (機場/gei1 coeng4/−基礎/gei1 co2/) or final-related pairs (法官/faat3 gun1/−奇觀/kei4 gun1/) as phonologically similar pairs. In contrast, this study used pairs where both characters are highly phonologically similar (安心/an1xin1/−安心/ansin/), which may result in a greater priming effect. Furthermore, although the behavioral results from Liu et al. (2023) suggested no significant difference in priming effects between phonologically similar and dissimilar cognate pairs, this does not mean that phonology was not activated during lexical recognition. In fact, the RTs under the phonologically similar condition were 57 ms shorter than those under the dissimilar condition (623 ms vs. 676 ms). The results of this study also indicate that phonological activation occurs relatively late (350–500 ms time window), almost at the same time as semantic activation, which made no significant difference in behavioral statistics.

### 4.3 Why is phonology limited in logographic script processing?

One potential explanation is that the morphological structure of logographic characters (such as Chinese characters) enables direct conveyance of semantic information. For example, the character “山” visually resembles a mountain, while the character “木” appears similar to a tree. Several empirical studies on Chinese character recognition indicate that when processing these characters, individuals first perceive structural attributes such as radicals, composition, and strokes, which relate to the semantic qualities of the character (McBride, 2016; Dulay et al., 2021). This morphological processing may exert a role in the early stages of recognition, suppressing the initial activation of phonological information. Additionally, the pronunciation of Chinese characters is contingent on context. An individual character may have distinct pronunciations and meanings across contexts, e.g., 单词 (dan1ci2: word) versus 单 (shan4: a surname) versus 单于 (chan2yu2: title for leaders of Xiongnu). In the absence of adequate contextual cues, there may be an increased tendency to first activate the semantics of the character, postponing phonological activation until sufficient contextual information has been obtained. This strategy could result in delayed activation of phonological information.

The experimental paradigms utilized also influence phonological activation. A key feature of the rapid masked priming paradigm is the extremely brief presentation time allotted for prime words. Such conditions may not facilitate the activation of phonological information, particularly for script systems such as Chinese, where the association between orthography and phonology is less robust compared to alphabetic scripts. Prior studies by Wong et al. (2014) and Zhou et al. (1999), employing Chinese two-character compound words as primes in rapid masked priming experiments, failed to uncover any phonological priming effects. This further intimates that under these experimental circumstances, observing phonological activation could prove challenging. However, in a study conducted by Zhang et al. (2019), utilizing single characters as experimental materials and expanding the prime word presentation duration to 140 ms, the obtained findings demonstrated that phonological activation transpired earlier than semantic activation. This implies that given adequate time, phonological information in Chinese can become activated, with this activation predating that of semantic information.

### 4.4 Theoretical implications

The findings from the current study offer insights pertinent to BIA+ models. Within the framework of the BIA+ model, input lexical information initially activates orthographic candidates, followed by activation of phonological, semantic, and additional lexical attributes. Phonology is activated preceding semantics, thereby expediting lexical recognition. However, the current work suggests that in recognition of logographic scripts, phonology and semantics are activated near-simultaneously, with interlingual phonological similarity failing to facilitate word processing. The characteristics of logographic scripts (like Chinese) recognition may provide a more nuanced contemplation and refinement of this model at the cognitive level.

The DevLex-II model (Zhao and Li, 2010; Zhao and Li, 2013) appears to offer a more comprehensive explanation for the findings of this study. DevLex-II model posits a significant difference in the lexical representation structure between early and late second language (L2) learners. According to this model, early L2 learners exhibit a distinct segregation of L2 lexical representations within the semantic map. In contrast, late L2 learners demonstrate a more constrained and fragmented spatial distribution of L2 lexical representations, which are interspersed among first language (L1) regions (Zhao and Li, 2010). Therefore, for late L2 learners, like those in this study who commence learning Japanese at the university level, their L2 representation is highly dependent on their L1 representation. When speakers come across a Japanese word, their initial response is to invoke the pronunciation of the Chinese cognate (instead of the Japanese pronunciation), which in turn enhances the processing of its meaning. Therefore, the similarity in phonology plays a minor role in their processing strategy.

## 5 Conclusion

In conclusion, the current study investigated whether phonological similarity elicits priming during the processing of logographic script (Chinese-Japanese) cognate pairs by manipulating the phonological similarity of such pairs. Masked priming lexical decision tasks showed no empirical evidence substantiating phonological facilitation, while ERP data indicated synchronous activation of phonological and semantic information during logographic script processing, revealing that in the processing of logographic scripts, the role of phonology is limited.

However, our conclusions may not extend to all circumstances, as there are unique cases in Chinese lexical processing. For example, Chinese contains “phonetic-semantic compounds” such as the character “漂,” where the phonetic component “票” relates to its pronunciation “piao1.” Given the common usage of “票,” its presence may enable more rapid activation of the phonology of “漂” compared to other non-phonetic-semantic compounds with comparable frequencies to “漂.” This phenomenon becomes more salient when the phonetic-semantic compound has low frequency. In other words, for low-frequency phonetic-semantic compounds, owing to the influence of the phonetic component, the process of phonological and semantic activation may not necessarily align with our findings. The activation of phonology could precede that of semantics. Additional empirical research is warranted to validate these speculative hypotheses.

## Data availability statement

The raw data supporting the conclusions of this article will be made available by the authors, without undue reservation.

## Ethics statement

The studies involving humans were approved by Ningbo University of Technology. The studies were conducted in accordance with the local legislation and institutional requirements. The participants provided their written informed consent to participate in this study.

## Author contributions

ZJ: Writing – original draft, Writing – review & editing, Conceptualization, Supervision. LD: Conceptualization, Data curation, Writing – review & editing. YW: Conceptualization, Data curation, Writing – review & editing. YL: Formal analysis, Visualization, Writing – review & editing.
